# Protective Effect of Ethanol Extracts of *Hericium erinaceus* on Alloxan-Induced Diabetic Neuropathic Pain in Rats

**DOI:** 10.1155/2015/595480

**Published:** 2015-04-16

**Authors:** Zhang Yi, Yang Shao-long, Wang Ai-hong, Sun Zhi-chun, Zhuo Ya-fen, Xu Ye-ting, He Yu-ling

**Affiliations:** ^1^Department of Endocrinology, Quanzhou First Hospital Affiliated to Fujian Medical University, Quanzhou, Fujian 362000, China; ^2^Department of Pathology, Zhengzhou Railway Vocational and Technical College, Zhengzhou, Henan 450000, China; ^3^Department of Endocrinology, 306 Hospital of PLA, Beijing 100101, China; ^4^Endocrine and Metabolic Diseases Division, First Affiliated Hospital, Guangxi Medical University, Nanning, Guangxi 530021, China

## Abstract

We investigated the effects of *Hericium erinaceus* (HEE) on alloxan induced diabetic neuropathic pain in laboratory rats. Alloxan induced diabetic rats were administered orally HEE. After 6 weeks of treatments, treatment with HEE 40 mg/kg in diabetic animals showed significant increase in pain threshold and paw withdrawal threshold and significant decrease in serum glucose and urine glucose. We also observed a significant increase in lactate dehydrogenase (LDH), Lipid peroxidation (LPO), glutathione peroxidase (GPx) activity, glutathione reductase (GR) activity, catalase (CAT) activity, Na^+^K^+^ATPase activity, and glutathione S transferase (GST) activity along with significant decreased levels of glutathione (GSH) content in diabetic rats. The total antioxidant status (TAOS) in the HEE-treated groups was significantly lower than that in the alloxan-treated group. HEE can offer pain relief in diabetic neuropathic pain. The improvement in diabetic state after HEE treatment along with the antioxidant activity could be the probable way by which it had alleviated diabetic neuropathy.

## 1. Introduction

One of the most common chronic complications of diabetes mellitus is diabetic neuropathy, which is mainly characterized by spontaneous pain and abnormal sensations such as paresthesia, allodynia, and hyperalgesia [1]. A large number of neuroanatomical, neurophysiological, and neurochemical mechanisms are thought to contribute to the development and maintenance of diabetic neuropathic pain (DNP) [2]. Current treatment of PDN involves the use of tricyclic antidepressant, selective serotonin reuptake inhibitors [3], anticonvulsants, opioids and antioxidant protein kinase C inhibitors, COX-2 inhibitors [4] and nonsteroidal anti-inflammatory drugs as mild analgesics, and so on. However, these therapies provide relief only to a fraction of patients and their side effect profiles limit their use [5, 6]. Therefore, there is a need to identify an effective and safe clinical treatment for PDN. Complementary medicines have gained in popularity among clinicians in recent years. Many indigenous medicinal plants/herbs have been found to be useful to successfully manage pain in various chronic pain models.

*Hericium erinaceus* is a mushroom that grows on old or dead broadleaf trees.* H. erinaceus* is taken as a food in Japan and China without harmful effects.* H. erinaceus* extracts and compounds have been found with special central effects that could be of pharmacological interest [7, 8]. However, the role of* H. erinaceus* in diabetic complications has not been investigated. The aim of the present investigation was to evaluate the neuroprotective effect of* H. erinaceus* against STZ-induced neuropathic pain and to assess its mechanism of action in rats.

## 2. Material and Methods

### 2.1. Preparation of* H. erinaceus* Extracts (HEE)

Fresh fruiting bodies of* H. erinaceus* were lyophilized and powdered. The dry powder (5 g) of mushrooms was extracted with 150 mL of ethanol for 2 h at room temperature, and* H. erinaceus* ethanol extract (499 mg) was obtained.

The extracts were stored at −30°C before use.

### 2.2. Animals

Healthy male adult Wistar rats (2 months old and weighing 225 ± 25 g) were used in the study. This study was performed in accordance with the Guide for the Care and Use of Laboratory Animals. Care was taken to minimize discomfort, distress, and pain to the animals.

### 2.3. Chemicals

Alloxan was purchased from Sigma (USA) and was dissolved in 0.1 N citrate buffers.

### 2.4. Experimental Design

Experimental diabetes was induced by a single intraperitoneal (i.p.) injection with alloxan (75 mg/kg) solution that was made with saline [9]. Forty-eight hours later, blood samples were collected from the tail veins of the rats. The blood glucose was analyzed with a Glucometer-4 (Bayer). Forty hyperglycemic rats (the blood glucose level greater than 11.1 mmol/L) were selected and allocated equally into 4 groups. From then on, the 4 groups of hyperglycemic rats were administered orally saline, HEE 10 mg/kg/d, HEE 20 mg/kg/d, and HEE 40 mg/kg/d, respectively. HEE was dissolved in the same amount of saline. The other 10 normal rats were administered orally with the saline and used as the control group. The serum glucose was measured before and at the end of the experiment to see the effect of pharmacological interventions on these parameters.

### 2.5. Assessment of Thermal Hyperalgesia

The nociceptive threshold was tested according to the Hargreaves procedure [10]. In brief, each animal was placed in a clear plexiglass box and the hind paw was exposed to a constant beam of radiant heat through a plexiglass surface. The time in seconds from initial heat source activation until paw withdrawal was recorded. HEE was administered in diabetic animals for 6 weeks, and withdrawal latency was noted daily 30 min after administration of HEE.

### 2.6. Assessment of Mechanical Allodynia

Mechanical allodynia was assessed before diabetes induction and subsequent to alloxan injection. In brief, each animal was placed in a test cage with a wire mesh floor, and the tip of a von Frey type filament was applied to the middle of the plantar surface of the hind paw and began to exert an upwards force until the paw was withdrawn or the preset cutoff was reached (40 g). A brisk foot withdrawal in response to von Frey type filament stimulation was recorded. The force required to elicit a withdrawal responses was measured in grams.

### 2.7. Estimation of Blood Glucose and Urine Glucose

The blood glucose was analyzed with a Glucometer-4 (Bayer). Urine glucose was assessed in fresh urine using glucose indicator sticks (Boehringer Mannheim, Germany).

### 2.8. Estimation of Antioxidant Enzymes Level

In serum, lactate dehydrogenase (LDH), glutathione (GSH) content, Lipid peroxidation (LPO), glutathione peroxidase (GPx) activity, glutathione reductase (GR) activity, catalase (CAT) activity, Na^+^K^+^ATPase activity, and glutathione S transferase (GST) activity were estimated using a method described by Lum et al. [11–17].

### 2.9. Estimation of Total Antioxidant Activity

The total antioxidant status (TAOS) of the supernatant of centrifuged plasma was determined by the way introduced by Laight et al. [18]. The increase in absorbance at 405 nm was measured by using a microplate reader (Shanghai Xunda Medical Technology, Inc., China).

### 2.10. Statistical Analysis

All data were analyzed by a one-way analysis of variance, and the differences between means were established by Duncan's multiple-range test. The data are shown as the mean ± SEM. The significant level of 5% (*P* < 0.05) was used as the minimum acceptable probability for the difference between the means.

## 3. Results

### 3.1. Effect of HEE Treatment on Thermal Hyperalgesia

**Figure 1:**
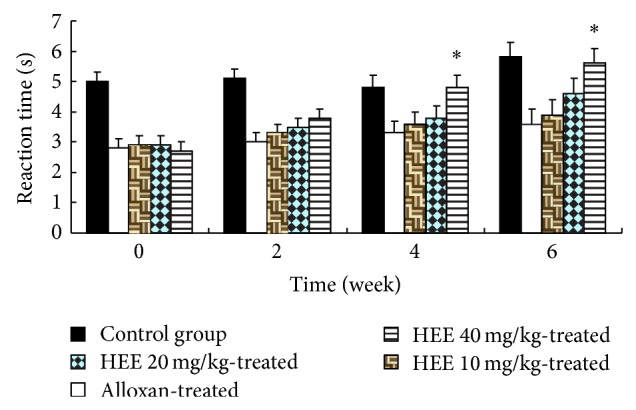
Effect of HEE treatment on tail withdrawal latency. Results are expressed as mean ± SEM (*n* = 10). The data was analysed using and one-way analysis of variance (ANOVA) followed by Dunnett's test. ^*^ *P* < 0.05 versus diabetic group.

Marked thermal allodynia was observed in the alloxan rats as evidenced by a reduction in the pain thresholds compared to control rats (Figure 1). Treatment of diabetic rats with HEE 40 mg/kg induced a significant increase in pain threshold compared to alloxan-treated animals after four weeks of treatment.

### 3.2. Effect of HEE Treatment on Mechanical Hyperalgesia

**Figure 2:**
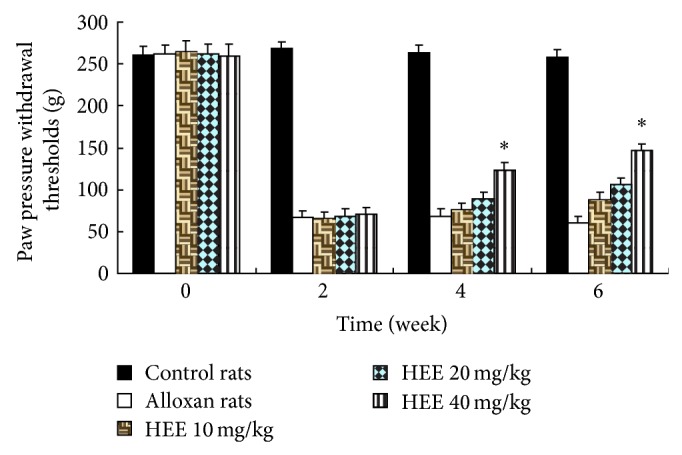
Effect of HEE treatment on paw withdrawal threshold. Results are expressed as mean ± SEM (*n* = 10). The data was analysed using and one-way analysis of variance (ANOVA) followed by Dunnett's test. ^*^ *P* < 0.05 versus diabetic group.

There was a marked mechanical hyperalgesia as evidenced by a reduction in the paw pressure withdrawal thresholds in the alloxan-treated animals (68.22 ± 8.47 g) compared to control-treated rats (266.14 ± 15.95 g). In rats receiving treatment of HEE 40 mg/kg mean paw withdrawal threshold was significantly and dose dependently increased compared to diabetic control rats (Figure 2).

### 3.3. Effect of HEE Treatment on Blood Glucose and Urine Glucose

The results of blood glucose and urine sugar from hyperglycemic rats are presented in Table 1. A significant decrease in the level of blood glucose and urine sugar were observed in HEE 40 mg/kg treated groups.

### 3.4. Effect of HEE Treatment on Antioxidant Enzymes Level

The serum LDH levels in control-treated rats were found to be 86.322 ± 2.663 IU/L. A significant increase in the activity of LDH in serum was observed in alloxan-treated animals. HEE 40 mg/kg significantly (*P* < 0.05) resulted in decreased serum LDH levels when compared with alloxan rats (Table 2). Also, HEE 40 mg/kg produced the increase in the level of GSH (Table 3). At the same time, a significant increase (*P* < 0.001) in the content of LPO was observed in the alloxan-treated animals. In the HEE group, a significant decrease (*P* < 0.01) was seen in the level of LPO when compared with the alloxan group (Table 4).

It has been proposed that antioxidant changes reflect an altered redox balance in several pathological states. Therefore, the measurement of endogenous antioxidants enzymes, that is, GPx, GR, CAT, and GST, as well as Na^+^K^+^ATPase, has been performed to estimate the amount of oxidative stress. Activities of various antioxidant enzymes and Na^+^K^+^ATPase of different groups have been listed in Table 5. HEE treatment showed a significant (*P* < 0.05–0.01) restoration in the level of various enzyme as compared with alloxan group.

### 3.5. Effect of HEE Treatment Total Antioxidant Activity

The results of TAOS are shown in Table 6. TAOS in the alloxan group were significantly (*P* < 0.01) higher than those in the control group. Those in the HEE-treated groups were significantly lower than those in the alloxan-treated group (*P* < 0.05).

## 4. Discussion

Diabetic neuropathy is characterized by clinical features like allodynia, hyperalgesia due to elevated nociceptive response. The present study indicates that alloxan-induced diabetes lowers pain threshold as evident by the presence of thermal hyperalgesia and mechanical allodynia. Although evaluation of mechanisms causing these symptoms is complicated because of the overlap between the systemic effects of hyperglycemia and its toxic effects within the peripheral nervous system, direct functional toxicity of hyperglycemia in the peripheral nervous system [19], administration of HEE after the 4 weeks of alloxan reversed diabetes induced thermal hyperalgesia (Figure 1) and mechanical allodynia (Figure 2). At the same time, treatment with HEE in diabetic animals showed significant decrease in serum glucose and urine sugar (Table 1). It indicates that the improvement on serum glucose levels may be attributed to the improvement on metabolic dysfunction in diabetic rats.

Hyperglycaemia-induced oxidative stress is an important mechanism leading to both the development and progression of hyperalgesia and allodynia in rats [20]. We observed a significant increase in LDH and reduction in endogenous antioxidant enzymes like GPx, GR, CAT, and GST as well as Na^+^K^+^ATPase activity along with significant decreased levels of GSH in diabetic rats. Treatment with HEE for six weeks restored the abovementioned biochemical parameters in diabetic rats in dose dependent manner. The TAOS is an indication of O_2_^−^ and other oxidant species. We also measured TAOS activity as an indirect indication of the formation of O_2_^−^ and other oxidant species. A marked increase in TAOS in diabetic animals has been observed in the present investigation. We also observed that HEE treatments dose-dependently attenuated oxidative stress, measured in terms of TAOS level, which may have further assist in management of painful diabetic neuropathy.

Collectively, administration of HEE could attenuate the diabetic neuropathy in rats. The improvement in diabetic state after HEE treatment along with the antioxidant activity could be the probable way by which it had alleviated diabetic neuropathy.

## Figures and Tables

**Table 1:** Effect of HEE on changes in the levels of blood glucose and urine sugar.

Groups	Blood glucose mmol/L	Urine sugar
Control group	5.04 ± 0.39	NIL
Alloxan group	8.08 ± 0.27	+++
HEE 10 mg/kg group	7.88 ± 0.20	+++
HEE 20 mg/kg group	7.03 ± 0.20	++
HEE 40 mg/kg group	5.26 ± 0.27^*^	NIL

Results are expressed as mean ± SEM (*n* = 10). The data was analysed using one-way analysis of variance (ANOVA) followed by Dunnett's test. ^*^ *P* < 0.05 versus diabetic group.

+++ indicates more than 2% sugar; NIL indicates 0 sugar; ++ and + indicate less than 2% sugar.

**Table 2:** Effect of HEE on serum LDH levels.

Different groups	LDH (IU/L)
Control group	80.322 ± 2.643^**^
Alloxan group	170.201 ± 3.441
HEE 10 mg/kg group	146.611 ± 2.221
HEE 20 mg/kg group	116.632 ± 3.221^*^
HEE 40 mg/kg group	130.230 ± 2.360^*^

Values are shown as means ± SEM. ^*^ *P* < 0.05 versus diabetic group and ^**^ *P* < 0.01 versus diabetic group.

**Table 3:** Effect of HEE on GSH.

Different groups	(nmol GSH/mg protein)
Control group	1.800 ± 0.016^*^
Alloxan group	1.011 ± 0.010
HEE 10 mg/kg group	1.090 ± 0.011
HEE 20 mg/kg group	1.500 ± 0.013^*^
HEE 40 mg/kg group	1.300 ± 0.011^*^

Values are shown as means ± SEM. ^*^ *P* < 0.05 versus diabetic group.

**Table 4:** Effect of HEE on LPO level.

Different groups	nmol
Control group	15.21 ± 0.66^**^
Alloxan group	21.20 ± 1.40
HEE 10 mg/kg group	20.00 ± 0.20
HEE 20 mg/kg group	17.36 ± 0.21^*^
HEE 40 mg/kg group	17.20 ± 0.20^*^

Values are shown as means ± SEM. ^*^ *P* < 0.01 versus diabetic group and ^**^ *P* < 0.001 versus diabetic group.

**Table 5:** Effect of HEE on the activity of various enzymes.

Different groups	GPx	GR	GST	CAT	Na^+^K^+^ATPase
Control	15.90 ± 1.20^***^	35.44 ± 2.50^***^	17.44 ± 1.00^**^	7.00 ± 0.55^*^	4.88 ± 0.31^**^
Alloxan	8.00 ± 0.41	20.55 ± 2.10	10.55 ± 1.44	4.33 ± 0.10	2.44 ± 0.11
HEE 10	9.11 ± 1.01	21.22 ± 2.00	10.66 ± 0.96	4.77 ± 0.21	2.00 ± 0.11
HEE 20	13.15 ± 1.33^**^	26.11 ± 2.11^***^	13.66 ± 0.90^*^	5.66 ± 0.33^*^	3.78 ± 0.10^*^
HEE 40	11.11 ± 1.10^*^	25.55 ± 2.35^**^	12.66 ± 0.77^*^	6.88 ± 0.38^*^	4.66 ± 0.11^*^

Values are shown as means ± SEM. ^*^ *P* < 0.05 versus diabetic group, ^**^ *P* < 0.01 versus diabetic group, and ^***^ *P* < 0.001 versus diabetic group.

**Table 6:** Effect of HEE on TAOS activity (*μ*M L-ascorbate).

Different groups	TAOS activity (*μ*M L-ascorbate)
Control group	28.41 ± 3.17^ **^
Alloxan group	80.33 ± 9.32
HEE 10 mg/kg group	72.22 ± 2.78^*^
HEE 20 mg/kg group	64.30 ± 3.38^*^
HEE 40 mg/kg group	56.35 ± 4.33^**^

Values are shown as means ± SEM. ^*^ *P* < 0.05 versus diabetic group and ^**^ *P* < 0.01 versus diabetic group.
